# Computed Tomography Assessment of Anatomic Graft Placement After ACL Reconstruction: A Comparative Study of Grid and Angle Measurements

**DOI:** 10.1177/2325967119832594

**Published:** 2019-03-19

**Authors:** Anagha P. Parkar, Miraude E.A.P.M. Adriaensen, Lasse M. Giil, Eirik Solheim

**Affiliations:** † Department of Radiology, Haraldsplass Deaconess Hospital, Bergen, Norway.; ‡ Department of Clinical Medicine, Faculty of Medicine and Dentistry, University of Bergen, Bergen, Norway.; § Department of Radiology, Zuyderland Medical Center, Heerlen, the Netherlands.; ∥ Department of Internal Medicine, Haraldsplass Deaconess Hospital, Bergen, Norway.; ¶ Department of Clinical Science, Faculty of Medicine and Dentistry, University of Bergen, Bergen, Norway.; *Investigation performed at Haraldsplass Deaconess Hospital, Bergen, Norway*

**Keywords:** anterior cruciate ligament, tunnel position, grid measurement, graft angles

## Abstract

**Background:**

The anatomic placement of anterior cruciate ligament (ACL) grafts is often assessed with postoperative imaging. In clinical practice, graft angles are measured to indicate anatomic placement on magnetic resonance imaging, whereas grid measurements are performed on computed tomography (CT). Recently, a study indicated that graft angle measurements could also be assessed on CT. No consensus has yet been reached on which measurement method is best suited to assess anatomic graft placement.

**Purpose:**

To compare the ability of grid measurements and angle measurements to identify anatomic versus nonanatomic tunnel placement on CT performed in patients undergoing ACL reconstruction.

**Study Design:**

Case series; Level of evidence, 4.

**Methods:**

A total of 100 knees undergoing primary reconstruction with a hamstring graft (HAM group), 91 undergoing reconstruction with a bone–patellar tendon–bone graft (BPTB group), and 117 undergoing revision ACL reconstruction (REV group) were assessed with CT. Grid measurements of the femoral and tibial tunnels and angle measurements of grafts were performed. Graft placement, rated as anatomic or nonanatomic, was assessed with both methods. Pearson chi-square, analysis of variance, Kruskal-Wallis, and weighted kappa tests were performed as appropriate.

**Results:**

The grid assessment classified 10% of the HAM group, 4% of the BPTB group, and 17% of the REV group as nonanatomic (*P* < .001). The angle assessment classified 37% of the HAM group, 54% of the BPTB group, and 47% of the REV group as nonanatomic. The weighted kappa between angle measurements and grid measurements was low in all groups (HAM: 0.009; BPTB: 0.065; REV: 0.041).

**Conclusion:**

The agreement between grid measurements and angle measurements was very low. The angle measurements seemed to overestimate nonanatomic tunnel placement. Grid measurements were better in identifying malpositioned grafts.

Anterior cruciate ligament (ACL) reconstruction has evolved constantly since it was implemented in clinical practice more than 3 decades ago.^8^ There have been many shifts in surgical trends, with the current focus mainly on anatomic reconstruction.^29^ Intraoperative and postoperative imaging is often used to assist and assess graft placement.^13,32,44^ Various imaging modalities and measurement methods are used to improve the reproducible assessment of postoperative graft placement.^31,41,47^ There is no consensus on which modality to use in clinical practice. The matter is further complicated by the fact that different measurement methods are used on the various radiological modalities for the assessment of femoral graft placement.^10,24,34^ The Bernard and Hertel grid is commonly used on radiographs and 3-dimensional computed tomography (3D-CT), with accurate measurements of graft placement in the high-low direction only possible on 3D-CT.^31^ There is less controversy regarding tibial graft placement, as measurements are performed in a similar manner in all modalities.^4,31,42^ The angle measurements of graft inclination or obliquity are mostly performed on magnetic resonance imaging (MRI).^3,4,27,38,46^

Studies have examined the normal anatomic locations of ACL insertions, which are used to judge postoperative placement as either “in” or “out” of the anatomic range.^1,9,11,19,25,34,35^ Several studies have also examined the normal angles of the native ACL.^3,5,36^ In clinical practice, it is common to perform MRI before primary surgery and revision to assess the soft tissues to detect/identify concomitant meniscal injuries and other ligament injuries. However, some also suggest the use of CT and 3D-CT to aid surgery planning before revision.^15,37^ In the clinical setting, it is not common to measure graft angles on CT or grids on MRI, and the methods are not used interchangeably. Recent studies show that there is little difference between angle measurements performed on MRI and CT and grid measurements performed on CT and 3D-MRI.^10,14^ However, it is not known if both methods are equally adept in identifying the nonanatomic placement of grafts.

The purpose of this study was to compare the angle and grid measurements of identifying anatomic versus nonanatomic tunnel placement on CT performed in patients undergoing ACL reconstruction. We hypothesized that the rate of nonanatomic placement would be higher in patients undergoing revision surgery compared with primary ACL reconstruction and aimed to assess the ability of the measurement methods to follow this hypothesis.

## Methods

The study was approved by a regional ethics board, and informed consent was waived. From January 2011 to the end of December 2017, all patients who were evaluated for revision ACL reconstruction and who underwent preoperative CT at a single institution were initially included retrospectively. Revision surgery was planned in patients who had an unsatisfactory function in the knee joint; examples of underlying causes were nonanatomic placement of graft tunnels, impingement due to grafts that were too long, or stretching of grafts during a new injury. Potential patients were identified through a manual search in our PACS system. From January 2011 to December 2015, we also included 100 postoperative CT scans obtained after primary reconstruction with either a hamstring graft or bone–patellar tendon–bone (BPTB) graft consecutively as separate groups. Postoperative CT was performed within 1 to 3 days after ACL reconstruction with the knee in full extension. Exclusion criteria were cases with multiple ligament reconstructions, known graft ruptures, or cases with previous revision. Graft ruptures were excluded, as graft angle measurements were not possible to perform without visible fibers on CT.

In all cases, sex, age, and knee laterality were recorded. In addition, the type of surgical technique (anteromedial portal or transtibial) and type of graft used (hamstring or BPTB) were recorded. In the revision group, months since primary ACL reconstruction were also recorded. All CT examinations were performed in our institution on either a 64- or 512-detector CT machine (GE Healthcare). All images were acquired at a tube voltage of 100 kV and tube current of 80 mA with a 0.625-mm slice thickness and reconstructed in 3 planes in soft kernel and bone algorithms with 2 mm–thick slabs.

**Figure 1.**
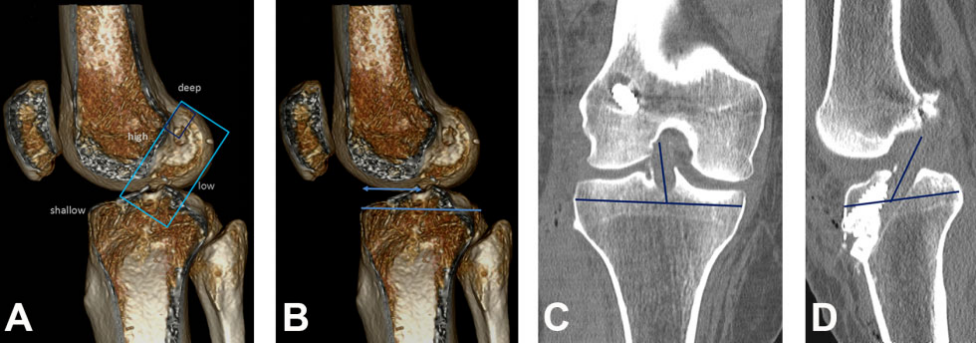
(A) Femoral tunnel measurement according to Bernard and Hertel, as depicted on 3-dimensional computed tomography (CT) after reconstruction with a hamstring graft. The graft tunnel center is 27% in the femoral deep-shallow direction and 35% in the femoral high-low direction (anatomic placement). (B) Tibial tunnel measurement according to Stäubli and Rauschning.^42^ The graft tunnel center is 46% in the tibial anterior-posterior direction (anatomic placement). (C) Coronal angle measurement on CT (example of reconstruction with a bone–patellar tendon–bone graft), measured at 80° (nonanatomic placement). (D) Sagittal angle measurement, measured at 60° (nonanatomic placement).

In all cases, measurements of femoral tunnel placement were performed according to Bernard and Hertel, tibial tunnel placement was assessed according to Stäubli and Rauschning,^42^ and ACL graft angles were measured in the coronal and sagittal planes. All measurements were performed and recorded by an experienced radiologist (>15 years; A.P.P.) (Figure 1). Normal ranges for the grid measurements were defined according to the literature: femoral deep-shallow, 24% to 37%; femoral high-low, 28% to 43%; and tibial anterior-posterior, 39% to 46%.^33^

The normal ranges for ACL coronal and sagittal angles were calculated from weighted means from the literature, as presented in Table 1.^3,4,36^ The normal coronal angle ranged from 66° to 74°, and the normal sagittal angle ranged from 47° to 59°. Graft placement in the coronal and sagittal planes was dichotomously recorded as “in” or “out” of the anatomic range. Within the revision group, the abovementioned analyses were also performed comparing the anteromedial portal and transtibial surgical approach.

**TABLE 1:** Normal Ranges of Coronal and Sagittal Graft Angles

	No. of Patients	Coronal Angle, deg	Sagittal Angle, deg
Ahn et al^3^ (2007)	50	65.9	58.7
Ayerza et al^5^ (2003)	30	–	51.0
Reid et al^36^ (2017)	188	74.3	46.9
Weighted mean (5th-95th percentile)		72.5 (66-74)	49.5 (47-59)

### Statistical Analysis

Dichotomous variables were assessed with the Pearson chi-square test. The combined assessments of grid in 2 directions or angles in 2 planes were classified in ordered categories (anatomic, partial anatomic, or nonanatomic), which were assessed with the weighted kappa. Continuous variables were assessed with the analysis of variance or Kruskal-Wallis test according to an assumed normality of data.^48^ *P* < .05 was considered significant, but the Bonferroni adjustment was used for multiple comparisons (*P* = .05, .017, or .08). All statistical analyses were performed with SPSS (v 25.0; IBM).

## Results

All of the primary ACL reconstructions were performed with the anteromedial portal approach. Within the study period, 137 patients were reviewed for revision surgery, of whom 6 were excluded from our study because of a ruptured graft and 14 were excluded because of multiple revisions. The final total of included revisions was 117 cases (REV group). Within the study period for primary ACL reconstruction, 100 cases of reconstruction with a hamstring graft (HAM group) and 91 cases of reconstruction with a BPTB graft (BPTB group) met the inclusion criteria.

There were statistically significant differences in the distribution of sexes between the 3 groups, with more female patients in the REV group (62%) compared with the HAM (45%; *P* = .01) and BPTB (44%; *P* = .008) groups. There was no difference between laterality and age between groups (Table 2).

**TABLE 2:** Demographics of Study Groups*^a^*

	Primary HAM (n = 100)	Primary BPTB (n = 91)	REV (n = 117)*^b^*	*P* Value
Age, y				.037 (K-W)
Mean ± SD	29 ± 10	26 ± 10	29 ± 9	
Median (range)	28 (14-54)	23 (14-53)	26 (15-55)	
Sex, n (%)				**.009** *^c^* (χ^2^)
Female	45 (45)	40 (44)	73 (62)	
Male	55 (55)	51 (56)	44 (38)	
Laterality, n (%)				.588 (χ^2^)
Right	53 (53)	42 (46)	61 (52)	
Left	47 (47)	49 (54)	56 (48)	

*^a^*Bolded *P* values indicate a statistically significant difference between groups (*P* < .05). BPTB, bone–patellar tendon–bone; HAM, hamstring; K-W, Kruskal-Wallis; REV, revision anterior cruciate ligament reconstruction.

*^b^*Months to revision surgery: mean ± SD, 50 ± 40; median (range), 36 (9-228).

*^c^*Pairwise (overall ***P* < .017**): HAM-BPTB: *P* = .88, HAM-REV: ***P*** **= .01**, BPTB-REV: ***P* = .008**.

### Grid Assessment

**Figure 2.**
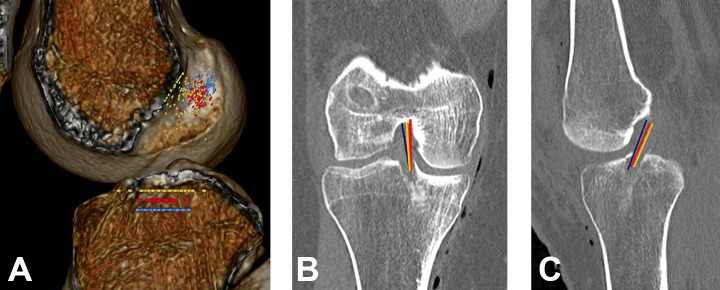
(A) Distribution of femoral and tibial tunnel placement between the 3 study groups. Differences in the mean (B) coronal angle and (C) sagittal angle between the 3 study groups. Blue line = hamstring; red line = bone–patellar tendon–bone; yellow line = revision anterior cruciate ligament reconstruction.

In both the pairwise comparisons of grid measurements and in the rates of anatomic versus nonanatomic placement, there were significant differences in the femoral deep-shallow measurement between the HAM and BPTB groups and between the HAM and REV groups (*P* < .001 for both), in which the mean graft placement in the REV group was shallower than in the HAM and BPTB groups (31% vs 24% and 28%, respectively). The nonanatomic rate in the HAM group was significantly worse than in the BPTB and REV groups (*P* ≤ .002 for both) (55% vs 20% and 34%, respectively). In the femoral high-low direction, for both the measurement and the rate of anatomic placement, there were significant differences between the HAM and REV groups and between the BPTB and REV groups (*P* < .001 for all). A significant difference was seen in the tibial measurement between the HAM and REV groups but not in the comparison of the rate of tibial nonanatomic versus anatomic placement. The rate of anatomic placement according to the combined grid assessment (anatomic, partial anatomic, and nonanatomic) differed significantly between the BPTB and REV groups but not between the HAM and BPTB groups or between the HAM and REV groups (Table 3 and Figure 2A).

**TABLE 3:** Results of Grid Measurements*^a^*

	Primary HAM (n = 100)*^b^*	Primary BPTB (n = 91)	REV (n = 117)	*P* Value
Femoral deep-shallow, %				**<.001** *^c^* (K-W)
Mean ± SD	24 ± 7	28 ± 5	31 ± 8	
Median (range)	24 (7-49)	28 (18-47)	30 (11-56)	
Graft placement, n (%)				**<.001** *^d^* (χ^2^)
Nonanatomic	55 (55)	18 (20)	40 (34)	
Anatomic	45 (45)	73 (80)	77 (66)	
Femoral high-low, %				**<.001** *^e^* (K-W)
Mean ± SD	28 ± 9	30 ± 7	20 ± 14	
Median (range)	29 (0-43)	30 (18-49)	20 (1-65)	
Graft placement, n (%)				**<.001** *^f^* (χ^2^)
Nonanatomic	45 (45)	36 (40)	84 (72)	
Anatomic	55 (55)	55 (60)	33 (28)	
Tibial, %				**.010** *^g^* (ANOVA)
Mean ± SD	46 ± 6	47 ± 4	49 ± 8	
Median (range)	46 (34-61)	48 (35-60)	48 (24-69)	
Graft placement, n (%)				.138 (χ^2^)
Nonanatomic	57 (58)	53 (58)	81 (69)	
Anatomic	42 (42)	38 (42)	36 (31)	
Combined grid assessment, n (%)				**<.001** *^h^* (χ^2^)
Nonanatomic	10 (10)	4 (4)	20 (17)	
Partial anatomic	79 (80)	67 (74)	89 (76)	
Anatomic	10 (10)	20 (22)	8 (7)	

*^a^*Bolded *P* values indicate a statistically significant difference between groups (*P* < .05). ANOVA, analysis of variance; BPTB, bone–patellar tendon–bone; HAM, hamstring; K-W, Kruskal-Wallis; REV, revision anterior cruciate ligament reconstruction.

*^b^*n = 99 for tibial and combined grid assessment.

*^c^*Pairwise (overall ***P* < .017**): HAM-BPTB: ***P*** **< .001**, HAM-REV: ***P*** **< .001**, BPTB-REV: *P* = .11.

*^d^*Pairwise (overall ***P* < .008**): HAM-BPTB: ***P*** **< .001**, HAM-REV: ***P*** **= .002**, BPTB-REV: *P* = .22.

*^e^*Pairwise (overall ***P* < .017**): HAM-BPTB: *P* = .413, HAM-REV: ***P*** **< .001**, BPTB-REV: ***P*** **< .001**.

*^f^*Pairwise (overall ***P <* .008**): HAM-BPTB: *P* = .447, HAM-REV: ***P*** **< .001**, BPTB-REV: ***P*** **< .001**.

*^g^*Pairwise (overall ***P* < .017**): HAM-BPTB: *P* = .620, HAM-REV: ***P*** **= .008**, BPTB-REV: *P* = .301.

*^h^*Pairwise (overall ***P* < .005**): HAM-BPTB: *P* < .09, HAM-REV: *P* = .412, BPTB-REV: ***P*** **< .001**.

### Angle Assessment

The coronal angle measurement differed significantly between the HAM and BPTB groups (*P* < .001) and between the BPTB and REV groups (*P* = .010), while the rate of anatomic versus nonanatomic placement differed between the HAM and BPTB groups (*P* = .001) and between the HAM and REV groups (*P* < .001). The sagittal angle measurement did not differ between the 3 groups, but the rate of anatomic placement differed significantly between the BPTB and REV groups. The combined angle assessment (anatomic, partial anatomic, and nonanatomic) did not differ significantly between groups (Table 4 and Figure 2, B and C).

**TABLE 4:** Results of Angle Measurements*^a^*

	Primary HAM (n = 100)*^b^*	Primary BPTB (n = 91)*^c^*	REV (n = 117)	*P* Value
Coronal angle, deg				**<.001** *^d^* (K-W)
Mean ± SD	72 ± 5	76 ± 5	74 ± 7	
Median (range)	72 (59-86)	77 (53-86)	75 (51-87)	
Graft placement, n (%)				**<.001** *^e^* (χ^2^)
Nonanatomic	44 (44)	61 (68)	81 (69)	
Anatomic	56 (56)	29 (32)	36 (30)	
Sagittal angle, deg				.019 (K-W)
Mean ± SD	65 ± 7	63 ± 5	62 ± 8	
Median (range)	64 (51-89)	62 (53-74)	63 (27-82)	
Graft placement, n (%)				**.011** *^f^* (χ^2^)
Nonanatomic	83 (83)	67 (74)	76 (65)	
Anatomic	17 (17)	24 (26)	41 (35)	
Combined angle assessment, n (%)				.137 (χ^2^)
Nonanatomic	37 (37)	49 (54)	55 (47)	
Partial anatomic	52 (52)	31 (34)	47 (40)	
Anatomic	11 (11)	11 (12)	15 (13)	

*^a^*Bolded *P* values indicate a statistically significant difference between groups (*P* < .05). BPTB, bone–patellar tendon–bone; HAM, hamstring; K-W, Kruskal-Wallis; REV, revision anterior cruciate ligament reconstruction.

*^b^*n = 100 for combined angle assessment.

*^c^*n = 91 for coronal angle and combined angle assessment.

*^d^*Pairwise (overall ***P* < .017**): HAM-BPTB: ***P*** **< .001**, HAM-REV: *P* = .061, BPTB-REV: ***P*** **= .010**.

*^e^*Pairwise (overall ***P* < .008**): HAM-BPTB: ***P*** **= .001**, HAM-REV: ***P*** **< .001**, BPTB-REV: *P* = .823.

*^f^*Pairwise (overall ***P* < .008**): HAM-BPTB: *P* = .115, HAM-REV: ***P*** **= .003**, BPTB-REV: *P* = .181.

### Agreement Between Measurement Methods

The overall agreement, assessed with the weighted kappa, between the grid and angle measurements to identify anatomic, partial anatomic, or nonanatomic was low. The pairwise agreement between groups was also low (Table 5).

**TABLE 5:** Comparison of Grid Versus Angle Measurements*^a^*

	Primary HAM (n = 100)*^b^*	Primary BPTB (n = 91)*^c^*	REV (n = 117)
Grid vs angle assessment (95% CI)			
Overall across groups		0.033 (–0.36 to 0.10)	
Weighted kappa within group	0.009 (–0.11 to 0.127)	0.065 (–0.39 to 0.169)	0.041 (–0.74 to 0.156)
Pairwise kappa			
HAM-BPTB		0.036 (–0.046 to 0.117)	
HAM-REV		0.032 (–0.52 to 0.115)	
BPTB-REV		0.046 (–0.035 to 0.128)	

*^a^*BPTB, bone–patellar tendon–bone; HAM, hamstring; REV, revision anterior cruciate ligament reconstruction.

^*b*^n = 99 for combined angle assessment.

^*c*^n = 91 for coronal angle and combined angle assessment.

### Comparison of Results Between Surgical Approaches

**Figure 3.**
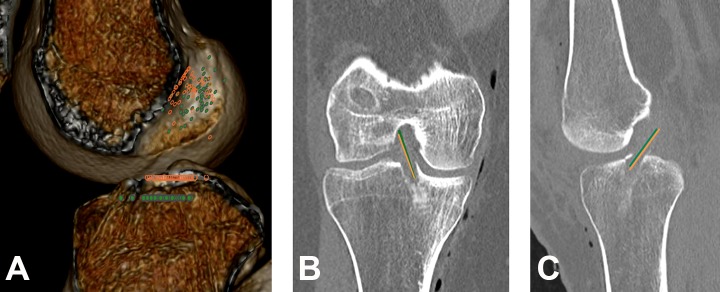
(A) Distribution of femoral and tibial tunnel placement in revision anterior cruciate ligament reconstruction. Differences in the mean (B) coronal angle and (C) sagittal angle in the revision group. Green line = anteromedial portal approach; orange line = transtibial approach.

Within the REV group, we further examined the differences between the transtibial and anteromedial portal approaches. In the femoral deep-shallow measurement and coronal angle measurement, there were no differences. In the femoral high-low measurement, tibial measurement, and sagittal angle measurement, there were significant differences (*P* > .001, *P* = .001, and *P* = .002, respectively). The rate of anatomic graft placement according to the combined grid assessment also differed between the surgical approaches. However, no differences were observed in the combined angle assessment with regard to anatomic versus nonanatomic placement (Table 3 and Figure 3). The agreement, assessed with the weighted kappa, between the grid and angle measurements to identify anatomic, partial anatomic, or nonanatomic placement was low in both the transtibial and anteromedial portal approach subgroups (Table 6).

**TABLE 6:** Comparison of Transtibial Versus Anteromedial Portal Approach Within REV Group*^a^*

	Anteromedial Portal (n = 66)	Transtibial (n = 51)	*P* Value
Femoral deep-shallow, %			.611 (K-W)
Mean ± SD	31 ± 9	31 ± 7	
Median (range)	31 (11 to 56)	30 (20 to 51)	
Graft placement, n (%)			.864 (χ^2^)
Nonanatomic	23 (35)	17 (33)	
Anatomic	43 (65)	34 (67)	
Femoral high-low, %			**>.001** (K-W)
Mean ± SD	24 ± 12	14 ± 15	
Median (range)	25 (0 to 45)	12 (–1 to 65)	
Graft placement, n (%)			**.001** (χ^2^)
Nonanatomic	39 (59)	45 (88)	
Anatomic	27 (41)	6 (12)	
Tibial, %			**.001** (K-W)
Mean ± SD	46 ± 8	52 ± 7	
Median (range)	47 (24 to 61)	52 (38 to 69)	
Graft placement, n (%)			**.021** (χ^2^)
Nonanatomic	40 (60)	41 (80)	
Anatomic	26 (40)	10 (20)	
Combined grid assessment, n (%)			**.004** (χ^2^)
Nonanatomic	10 (15)	10 (20)	
Partial anatomic	48 (73)	41 (80)	
Anatomic	8 (12)	0 (0)	
Coronal angle, deg			.398 (K-W)
Mean ± SD	73 ± 7	74 ± 6	
Median (range)	75 (53 to 87)	75 (52 to 86)	
Graft placement, n (%)			.082 (χ^2^)
Nonanatomic	50 (75)	31 (61)	
Anatomic	16 (25)	20 (39)	
Sagittal angle, deg			**.002** (K-W)
Mean ± SD	60 ± 8	65 ± 8	
Median (range)	60 (27 to 73)	65 (49 to 82)	
Graft placement, n (%)			**.022** (χ^2^)
Nonanatomic	37 (56)	39 (76)	
Anatomic	29 (44)	12 (34)	
Combined angle assessment, n (%)			.639 (χ^2^)
Nonanatomic	29 (44)	26 (51)	
Partial anatomic	29 (44)	18 (35)	
Anatomic	8 (12)	7 (14)	
Grid vs angle assessment (95% CI)			
Weighted kappa within approach	0.074 (–0.82 to 0.23)	–0.006 (–0.86 to 0.163)	
Overall across both approaches	0.041 (–0.74 to 0.156)		

*^a^*Bolded *P* values indicate a statistically significant difference between approaches (*P* < .05). K-W, Kruskal-Wallis; REV, revision anterior cruciate ligament reconstruction.

## Discussion

The purpose of our study was to compare grid measurements and angle measurements with regard to the anatomic placement of grafts after ACL reconstruction. The major finding of our study was the lack of agreement between the 2 measurement methods in identifying anatomic graft placement.

**Figure 4.**
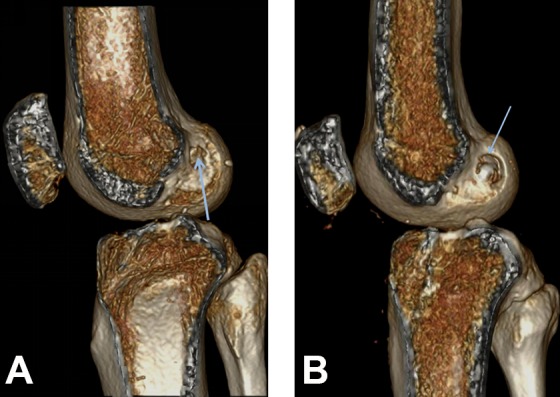
(A) The graft tunnel aperture in reconstruction with a hamstring graft; the aperture center is the same as the graft center (arrow). (B) The graft tunnel aperture looks larger in reconstruction with a bone–patellar tendon–bone graft, but the graft is actually placed slightly deeper than the aperture center (arrow).

Technical errors, such as nonanatomic graft placement, are considered a common cause of graft failure, so one would expect the rate of nonanatomic placement to be higher in patients with failed ACL grafts.^28,30^ The literature suggests that femoral tunnel placement is more important for a favorable outcome compared with tibial tunnel placement.^6,28^ The results of the grid assessment confirmed this assumption, with no difference between the 3 groups in tibial tunnel placement but a difference in the overall femoral tunnel placement. Placement in the femoral deep-shallow direction did not differ significantly between the BPTB and REV groups. This may be explained by the fact that the tunnel aperture on CT in the BPTB group may not represent the true center of the graft, as the bony attachment is a few millimeters thick, thus slightly influencing the grid measurement (Figure 4). Surprisingly, the rate of nonanatomic placement in the femoral deep-shallow direction was highest in the HAM group. In the femoral high-low direction, the nonanatomic placement rate was significantly higher in the REV group compared with the HAM and BPTB groups, as was to be expected. This finding might indicate that anatomic placement in the high-low direction in the femur is more important for outcomes than anatomic placement in the deep-shallow direction and that the surgical technique should aim to avoid nonanatomic placement in the high-low direction.

No difference in the groups was observed in tibial graft placement. Regarding overall nonanatomic graft placement, the highest rate was observed in the REV group (17% in REV vs 10% in HAM and 4% in BPTB). Pairwise comparisons showed no difference between the HAM and BPTB groups, as was expected. However, there was also no difference between the HAM and REV groups. This may be explained by the high rate of nonanatomic placement in the HAM group in the femoral deep-shallow direction, influencing the overall anatomic rate in the grid measurements. There was a significant difference in the combined grid assessment between the BPTB and REV groups.

Angle measurements for assessing postoperative graft placement are often recommended in the literature.^12,13,30,47^ In the coronal measurements, we found significant differences between the HAM and BPTB groups and between the BPTB and REV groups. In general, in the BPTB group, we observed a steeper (nonanatomic) coronal angle than in the other groups. The reason for this may again be the bony attachment of the BPTB graft, which when placed caudally in the tunnel will cause a more cranial exit for the tendon, causing it to run a steep slope in the coronal view. There were no differences in the sagittal angle measurements or in the combined angle assessment for anatomic placement between groups. The highest rate of overall nonanatomic placement was seen in the BPTB group (54% in BPTB vs 37% in HAM and 47% in REV).

The 2 grid and the angle methods yielded significantly different rates of nonanatomic placement in the same patients. The explanation for this is a fundamental difference in measurement methods. The angle measurements were devised in the era of the transtibial surgical technique. Technical failure with the transtibial technique was related to “too high” placement of the femoral tunnel and was easily assessed on sagittal images, and a too steep graft angle was introduced as an imaging criterion.^3,39,45^ However, it is known that flexion affects the ACL angle in the sagittal plane. A study showed that the sagittal angle of the ACL ranges from 45° to 20° with increasing knee joint flexion.^16^ This factor affects the measurements in a clinical setting, as even the slightest flexion during CT or MRI will change the angle of the ACL graft. Furthermore, even if the angle is correct, the placement may still be faulty if the graft is placed too anteriorly or too posteriorly.^32^ The grid measurements were devised so that the measurements in the femur and tibia are independent of the degree of knee flexion. Thus, methodological discrepancies relating to knee flexion may explain the poor agreement between the 2 measurement methods. CT is the more robust modality if one chooses to measure graft placement.

Considering revision ACL reconstruction, previous studies have shown that the anteromedial portal technique yields higher rates of anatomic placement compared with the transtibial technique.^39,45^ The transtibial technique is known to cause too high placement in the grid measurements, which was confirmed in our study.^17,43^ The combined grid assessment differed significantly between the surgical techniques (*P* = .004), with no anatomic cases in the transtibial technique group. In addition, the sagittal angle differed between the surgical techniques (*P* = .002), while the coronal angle measurements and combined angle assessment did not differ within the REV group. Thus, the lack of agreement between the grid and angle measurement methods was also observed within the REV group.

The clinical usefulness of (1) postoperative CT in primary reconstruction to improve a surgeon’s learning curve and to serve as a baseline examination and (2) preoperative CT for planning revision surgery has been established.^20,40,49^ MRI undoubtedly has a role in planning revision surgery for identifying recurrent ACL graft ruptures and missed concomitant lesions in other ligaments, menisci, or articular cartilage.^2,22,37^ Ducouret et al^10^ found that angle measurements did not differ between CT and MRI and suggested that MRI can be used to replace CT for identifying tunnel placement. Grasso et al^14^ performed grid measurements on CT and MRI; however, the measurements were conducted on computer-generated models after adding digitized information acquired during revision surgery, not on actual CT or MRI scans. In our view, the clinical usefulness of angle and grid measurements on MRI has not been sufficiently established to date. No studies have examined the clinical benefit of angle measurements in reconstruction using the anteromedial portal technique. Furthermore, our results show that graft angles do not correlate with grid measurements, and they overestimate nonanatomic placement in ACL reconstruction with the anteromedial portal technique. Therefore, MRI currently cannot replace CT to identify anatomic graft placement in ACL reconstruction.

This study highlights the problems that arise because of the lack of consensus on which measurement method to use when assessing ACL graft placement. Several studies have compared graft placement between the transtibial and anteromedial portal techniques, but the studies used several different measurement methods to assess graft placement.^7,21,26,46^ This makes a comparison of surgical results difficult, as we now know that the reported rate of nonanatomic tunnel placement varies depending on the method used.

This is the first study comparing grid and angle measurement methods after ACL reconstruction. We have laid bare the major discrepancy between these methods. As previous studies have shown low interrater and intrarater variability in both methods used in our study, we did not assess interrater variability and do not consider this a major limitation.^18,23^ The normal ranges of grid and angle measurements are based on a relatively high number of anatomic and imaging cases (>200-300).^3,5,33,36^ This limits bias in identifying the appropriate cutoff in our study. As the purpose of our study was to compare 2 methods used for assessing anatomic tunnel placement on imaging, we did not correlate graft placement with clinical or functional assessments of graft laxity and cannot determine whether nonanatomic tunnel placement affects graft laxity.

## Conclusion

The agreement between angle and grid measurements to identify anatomic ACL graft placement was very low. Compared with grid measurements, angle measurements tended to overestimate nonanatomic tunnel placement. Grid measurements were better in identifying malpositioned ACL grafts. Orthopaedic surgeons and radiologists ought to be aware of the pitfalls of the angle measurement method when assessing ACL graft placement on imaging.
